# Soluble suppression of tumorigenicity-2 changes during cardiotoxic cancer treatment: a systematic review and meta-analysis

**DOI:** 10.3389/fcvm.2025.1624023

**Published:** 2025-11-11

**Authors:** Luca Fazzini, Simone Angius, Nicola Campana, Luca Pascalis, Martino Deidda, Giordano Maria Pugliesi, Vincenzo Quagliariello, Nicola Maurea, Carlo Gabriele Tocchetti, Pietro Ameri, Christian Cadeddu Dessalvi

**Affiliations:** 1 Department of Medical Sciences and Public Health, University of Cagliari, Cagliari, Italy; 2 Department of Cardiovascular Medicine, Mayo Clinic, Rochester, MN, United States; 3 Department of Cardiovascular Medicine, University of Milano-Bicocca, Milan, Italy; 4 Division of Cardiology, Istituto Nazionale Tumori-IRCCS-Fondazione G. Pascale, Napoli, Italy; 5 Internal Medicine Unit for Cancer Patients, Department of Translational Medical Sciences (DISMET), Federico II University, Naples, Italy; 6 Center for Basic and Clinical Immunology Research (CISI), Federico II University, Naples, Italy; 7 Interdepartmental Center for Clinical and Translational Research (CIRCET), Federico II University, Naples, Italy; 8 Interdepartmental Hypertension Research Center (CIRIAPA), Federico II University, Naples, Italy; 9 Cardiovascular Disease Unit, IRCCS Ospedale Policlinico San Martino, Genoa, Italy; 10 Department of Internal Medicine, University of Genova, Genova, Italy

**Keywords:** cardioncology, biomarker, ST2, cardiotoxcity, cardiotoxic adverse effect

## Abstract

**Background:**

Soluble suppression of tumorigenicity-2 (sST2) is a promising biomarker of cardiovascular disease and heart failure. Data about the changes in sST2 concentrations during cancer treatment and the relationship with cancer treatment-related cardiotoxicity are sparse.

**Methods:**

We conducted a systematic review and meta-analysis to explore longitudinal changes in sST2 levels at three time points (T0 baseline, T1 post-chemotherapy, and T2 follow-up) in cancer patients treated with cardiotoxic therapies and compared these changes to traditional biomarkers of cardiac injury, i.e., troponin and NT-proBNP. Using random-effects models, mean differences (MD), and standardized MD (SMD), we analyzed (i) ST2 longitudinal changes, (ii) the association between ST2 and cardiotoxicity [defined through left ventricular ejection fraction (LVEF)] providing pooled estimates of correlations, and (iii) the SMD variations among biomarkers.

**Results:**

Eight studies were included, comprising 433 patients treated with anthracycline and/or HER2-directed antibodies. There was a trend toward increased sST2 levels from T0 to T2 (MD 1.86, 95% CI −0.97 to 4.68, *p* = 0.200) and decreased levels from T1 to T2 (MD −1.96, 95% CI −4.28 to 0.37, *p* = 0.100). A pooled analysis showed a negative correlation between sST2 levels and LVEF (r −0.29, 95% CI, −0.49- −0.05, *p* < 0.010). Comparisons with Troponin and NT-proBNP showed a significantly higher Troponin SMD at T0-T1 (*p* = 0.027), while no significant differences were observed for NT-proBNP.

**Conclusion:**

sST2 showed dynamic changes during cardiotoxic therapy correlating with cardiotoxicity. Troponin was demonstrated to have greater longitudinal variations. Further research is needed to evaluate longitudinal sST2 levels in patients who develop cardiotoxicity vs. those who do not.

## Introduction

Despite the impact of new-generation anti-cancer therapies on contemporary cancer management, cardiotoxic agents, such as anthracyclines, remain a cornerstone in the treatment of solid and hematological cancers (1). Advances in early cancer diagnosis and treatment have turned some cancers from fatal illnesses into manageable chronic conditions (2). However, improved survival rates are often accompanied by treatment-related complications, ultimately leading to increased cardiovascular mortality (3, 4). Given the life-saving nature of these treatments, their use is often indispensable, making it crucial to focus on the screening, monitoring, and management of preclinical and clinical cancer therapy-related cardiac dysfunction before the development of overt heart failure (HF).

Recently, significant attention has been directed toward identifying patients at elevated risk for cardiotoxicity. Baseline cardiovascular risk factors have been recognized as important predictors, eventually leading to the development of tools like the HFA-ICOS score, as highlighted in the latest guidelines (5–8). However, these risk factors alone cannot fully characterize and identify the high-risk population. In this context, sensitive biomarkers have proven invaluable in detecting cardiotoxicity at an early stage, with Troponin and NT-proBNP being the most extensively studied and recommended (9–11).

Within HF, a variety of biomarkers have been identified for diagnostic and prognostic purposes, encompassing myocardial injury biomarkers, neurohormonal markers, and those related to inflammation, fibrosis, genetics, metabolism, and genomics (12–14). Among these, the soluble suppression of tumorigenicity-2 (sST2) has emerged as a novel biomarker. sST2 is the circulating form of the cellular receptor for interleukin-33, serving as an indicator of cardiac remodeling and prognosis in HF (15–17). While the role of sST2 in HF is well established, its potential for monitoring chemotherapy-induced cardiotoxicity remains largely underexplored.

This meta-analysis aims at evaluating the available evidence on the role of sST2 in monitoring chemotherapy-related cardiac injury, offering insights into its applicability and analyzing the relationship between sST2 and traditional biomarker's longitudinal changes.

## Methods

### Eligibility criteria

Inclusion in this meta-analysis was restricted to studies that met all the following eligibility criteria: (1) enrolled patients with cancer treated with cardiotoxic therapies; (2) reported sST2 specific measurements at baseline; (3) reported longitudinal sST2 measurements after the baseline measurement either at the end of the cardiotoxic therapy administration or at 3–6 months follow-up. We excluded studies written in languages other than English.

### Search strategy and data extraction

We systematically searched PubMed, and Scopus from inception to December 2024 with the following search terms: 'sST2', 'ST2', “anthracycline”, “cardiotoxicity”, “cardio-oncology”, “cardiovascular toxicity”, “cardiac toxicity”, and “chemotherapy-induced cardiotoxicity”. The detailed search strategy is reported in Supplementary Table S1. The references from all included studies and previous systematic reviews were also searched manually for any additional studies. The literature obtained from the searches was independently filtered by two authors (L.F. and S.A.) using titles, abstracts, and full text when deemed appropriate. They independently extracted the data following predefined search criteria in a dedicated electronic database. Any discrepancies and disagreements were resolved by the senior author (C.C.D.).

### Objectives

The primary objective is to describe the sST2 longitudinal changes in cancer patients treated with cardiotoxic therapies. Secondly, we aim at combining the correlations between sST2 and the development of cardiotoxicity that each study reported in its result section to provide an estimated overall correlation. Supplementary Table S2 reports the definitions of cardiotoxicity provided by each study. Finally, we aim to compare the sST2 to the traditional biomarkers in terms of longitudinal changes.

### Statistical analysis

This systematic review and meta-analysis was performed in accordance with the Cochrane Collaboration and the Preferred Reporting Items for Systematic Reviews and Meta-Analysis (PRISMA) statement guidelines (18). Biomarker measurements were evaluated longitudinally at three time points: T0 (baseline), T1 (post-chemotherapy completion), and T2 (3–6 months of follow-up). In this meta-analysis, we utilized the mean difference (MD) as the effect measure to quantify changes in sST2 levels between T0, T1 and T2, among patients exposed to cardiotoxic therapies. The choice of MD was based on the uniformity of measurement units and scales for sST2 across the included studies, allowing for direct comparison of absolute differences. To account for potential heterogeneity among studies, arising from variations in patient populations, study protocols, and follow-up durations, we employed a random-effects model. This approach assumes that the true effect size may vary across studies and provides a more conservative estimate, thus enhancing the generalizability of the findings. The common-effects model is presented in the forest plots as a sensitivity analysis. Given that biomarker measurements are continuous outcomes, they were transformed to mean and standard deviation by the method proposed by Wan et al. when needed (19). Subsequently, the restricted maximum-likelihood estimator (REML) method was applied to reduce variance bias, and the MD among T0, T1, and T2 was computed (20). Cochran Q test and *I*^2^ statistics were used to assess heterogeneity; *P* values inferior to 0.10 and *I*^2^ > 25% were considered significant for heterogeneity.

Subsequently, we conducted a meta-analysis to combine correlation coefficients (r) reported in studies exploring the relationship between sST2 and cardiotoxicity. The correlation coefficient (r) and the number of patients were extracted from each study. For studies reporting no correlation explicitly, a value of r = 0 was assumed to be “0”, the most conservative value. To stabilize variance and improve the normality of the correlation coefficients, we applied Fisher's transformation (21). Results were visualized using a forest plot, where each study's r, sample size, and confidence interval were displayed alongside the pooled correlation coefficient.

Finally, to compare the performance of sST2, Troponin, and NT-proBNP in detecting changes from T0 to T1 and T2, given that the units of measure of NT-proBNP and Troponin were heterogeneous among studies, we calculated the Standardized Mean Difference (SMD) for each biomarker across studies (22). A two-sample t-test was applied to evaluate whether the mean SMD of sST2 was significantly different from the mean SMD of Troponin and NT-proBNP. This approach treats each SMD value derived from individual studies as an independent observation and compares the overall distributions of SMDs between the two biomarkers. The test provides a single *p*-value reflecting the overall difference between the two biomarkers' longitudinal changes. The data is then visualized using a line plot, where each study is represented by a pair of points corresponding to the SMD of sST2 and either Troponin or NT-proBNP. The plot includes lines connecting these points for each study, highlighting the change between the two biomarkers.

All statistical analyses were performed using R (R Foundation for Statistical Computing, Vienna, Austria; version 4.3.2) within RStudio.

## Results

### Study selection and characteristics

Supplementary Figure S1 shows the PRISMA flowchart for study selection. The initial search yielded 59 results. After the removal of duplicate records and ineligible studies, 16 manuscripts were fully reviewed based on inclusion criteria. Of these, a total of 8 studies were included, comprising 433 patients.

The study and baseline characteristics of the included subjects are reported in Table 1. The median age ranged from 42 to 57 years. Six studies enrolled only women with breast cancer, while two studies enrolled 61% and 83% women with breast cancer accounting for 46% and 67% of enrolled patients, respectively. All the studies included patients treated with anthracyclines and/or HER-2 antibodies. Given the relevance of cardiovascular risk factors in high-risk individuals identification, we reported hypertension, dyslipidemia, and diabetes prevalence in Table 1.

**Table 1:** Study and baseline characteristics.

First Author	Year	Sample size, (n)	Age (y)^a^	Female (%)	Breast Cancer (%)	Chemotherapy Regimen	Hypertension *n* (%)	Dyslipidemia *n* (%)	Diabetes *n* (%)
Bhaghat (27)	2023	31	50 (46–55)	100	100	Anthracyclines	10 (32)	7 (23)	2 (7)
Dean (29)	2023	41	55.7 (15.7)	61	46	Anthracyclines	11 (27)	NA	4 (10)
Gherghe (37)	2022	22	55.6 (9.9)	100	100	HER2 antibody	4 (18)	6 (27)	3 (14)
Huang (38)	2018	126	53.3 (5.0)	100	100	Anthracyclines or HER2 antibody or combined	31 (25)	22 (17)	16 (13)
Isemede (39)	2022	81	57 (48–64)	100	100	Anthracyclines	NA	NA	NA
Rosenkaimer (40)	2022	21	55.8 (13.5)	100	100	Anthracyclines or HER2 antibody or combined	8 (38)	15 (71)	4 (19)
Sawaya (28)	2012	81	50 (10)	100	100	Anthracyclines or HER2 antibody or combined	26 (32)	18 (22)	1 (1)
Shirzadi (41)	2021	30	42.3 (10.6)	83	67	Anthracyclines	NA	NA	NA

a Age is reported as described by each study. Either mean and standard deviation or median and interquartile ranges.

### sST2 longitudinal changes

sST2 was measured by different assays among studies, which are reported in Supplementary Table S3. All studies reported sST2 baseline values. Four studies reported sST2 values after chemotherapy completion. Seven studies reported sST2 values at follow-up. Among these, six studies had 6-month follow-up, while one study had 3–6-month follow-up. The absolute values of sST2 across the three time points (T0, T1, and T2) are summarized in Table 2.

**Table 2:** Absolute values of sST2 at baseline (T0), after chemotherapy (T1), and at follow-up (T2).

First Author	sST2 baseline (T0)^a^	sST2 after chemotherapy (T1)^a^	sST2 at follow-up (T2)^a^	Follow-up duration (months)
Bhaghat	21.3 (15.6, 24.9)	24.1 (18.6, 30.8)	22.2 (17.8, 26.4)	6
Dean	24.0 (17.4–31.4)	24.2 (17.7–37.6)	20.2 (15.7–25.8)	3 to 6
Gherghe	24.2 (20.7)	NA	23.3 (26.7)	6
Huang	6.5 (1.3)	NA	13.0 (4.7)	6
Isemede	27.3 (22.8–32.8)	26.1 (20.2–36.4)	NA	NA
Rosenkaimer	20.2 (9.5)	NA	24.8 (11.2)	6
Sawaya	26 (23–35)	27 (23–42)	26 (21–32)	6
Shirzadi	42.8 (0.8)	NA	45.6 (1.9)	6

a The unit of measure is ng/ml. Values are reported as described by each study. Either mean and standard deviation or median and interquartile ranges.

**Figure 1:**
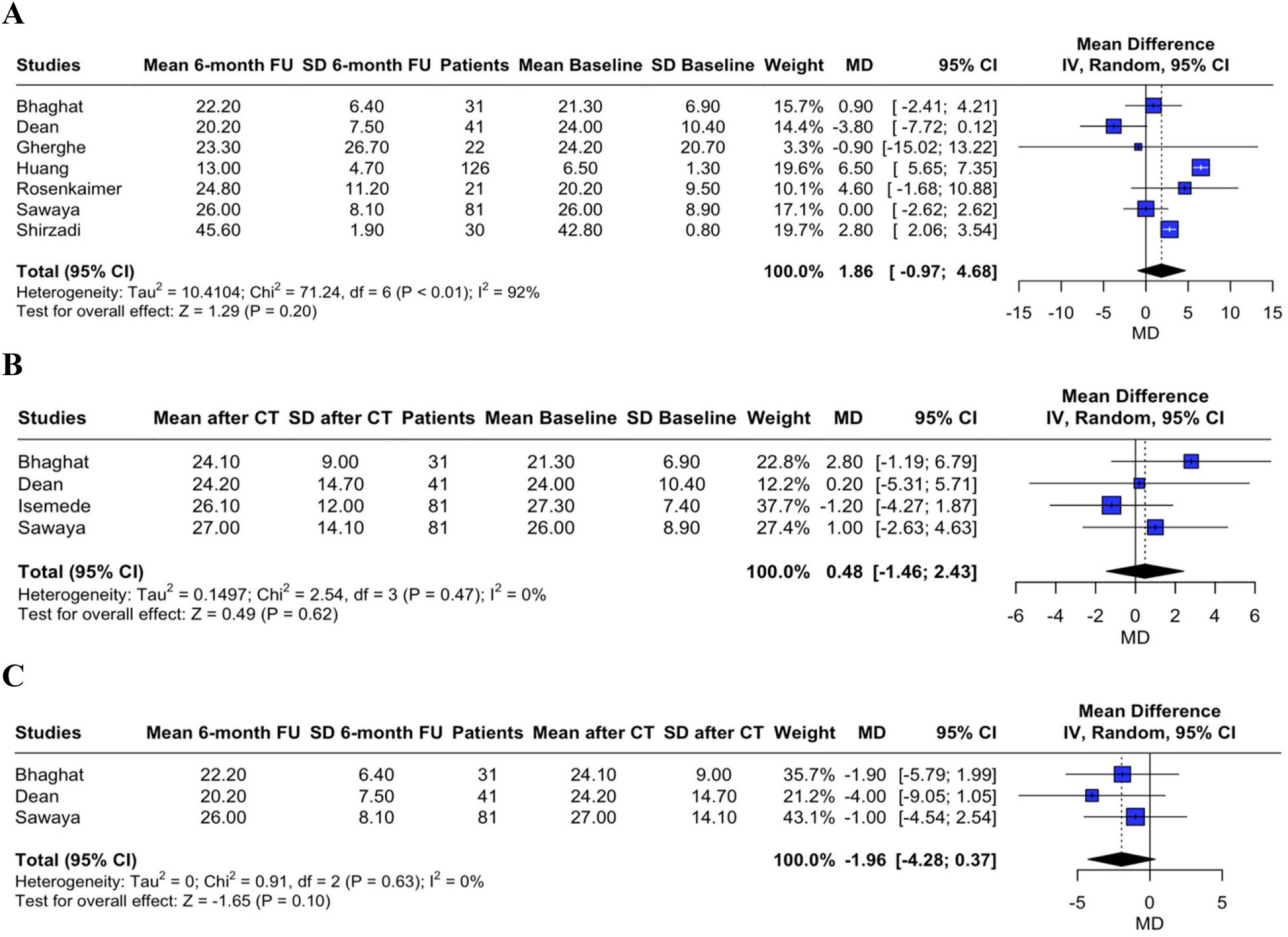
Forest plot showing the sST2 mean difference between T0 and T2 (panel **A**), between T0 and T1 (panel **B**), and between T1 and T2 (panel **C**).

sST2 level changes were assessed as the MD between the three time points. A non-significant trend was found towards increased sST2 levels during cardiotoxic therapy administration and towards decreased sST2 levels at follow-up, as compared to sST2 levels after chemotherapy completion (MD between T0 and T2 1.86, 95% CI, −0.97–4.68, *p* = 0.200, *I*^2^ = 92%, Figure 1A; MD between T0 and T1 0.48, 95% CI, −1.46–2.43, *p* = 0.620, *I*^2^ = 0%, Figure 1B; MD between T1 and T2 −1.96, 95% CI, −4.28–0.37, *p* = 0.100, *I*^2^ = 0%, Figure 1C**)**.

**Figure 2:**
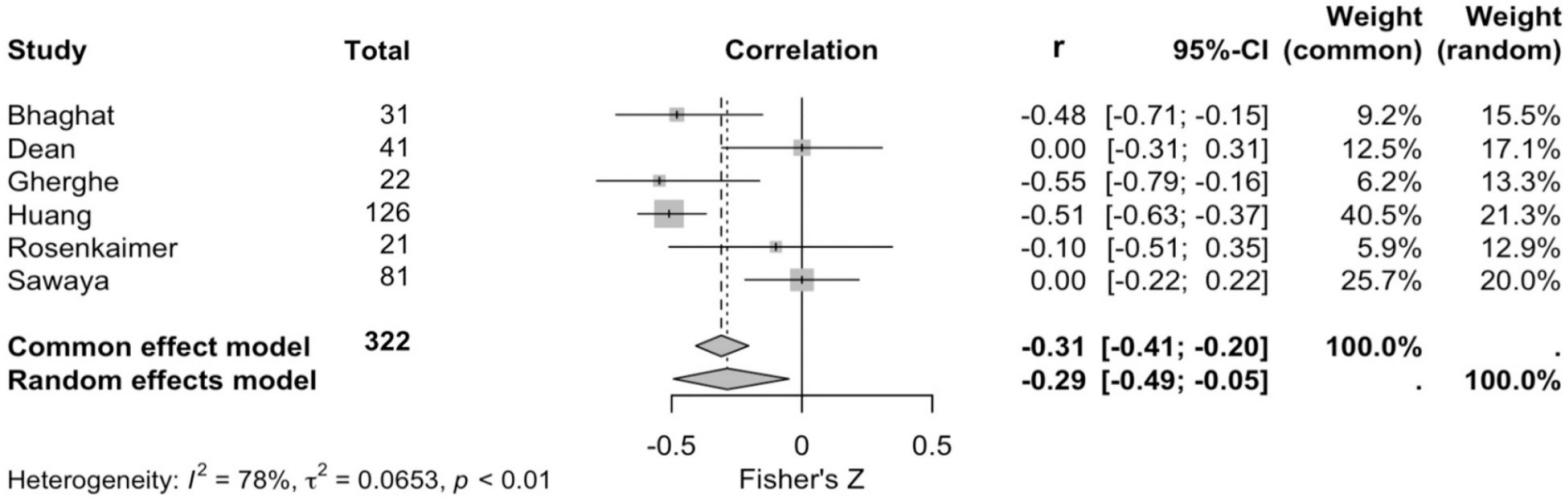
Forest plot showing the meta-analysis of correlations between sST2 and cardiotoxicity.

Six studies explored the correlation between sST2 and cardiotoxicity development. Cardiotoxicity definitions are reported in Supplementary Table S2. According to a random effects model (and common effects model as a sensitivity analysis), there was a negative correlation between sST2 levels and left ventricular ejection fraction (r −0.29, 95% CI, −0.49- −0.05, *p* < 0.010, *I*^2^ = 78%, Figure 2).

### Comparison between sST2, troponin, and Nt-proBNP longitudinal changes

**Figure 3:**
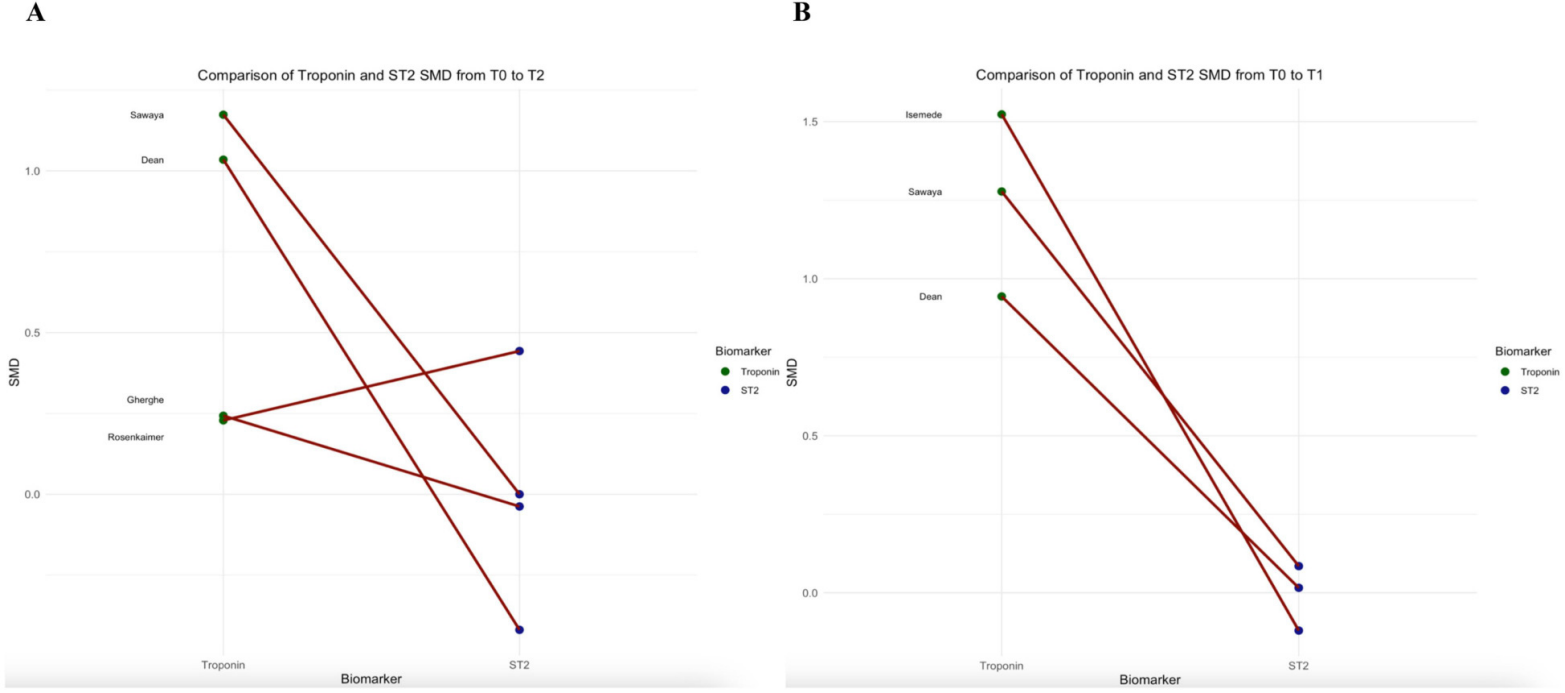
Comparison of troponin and sST2 standard mean differences between T0 and T2 (panel **A**), and T0 and T1 (panel **B**).

**Figure 4:**
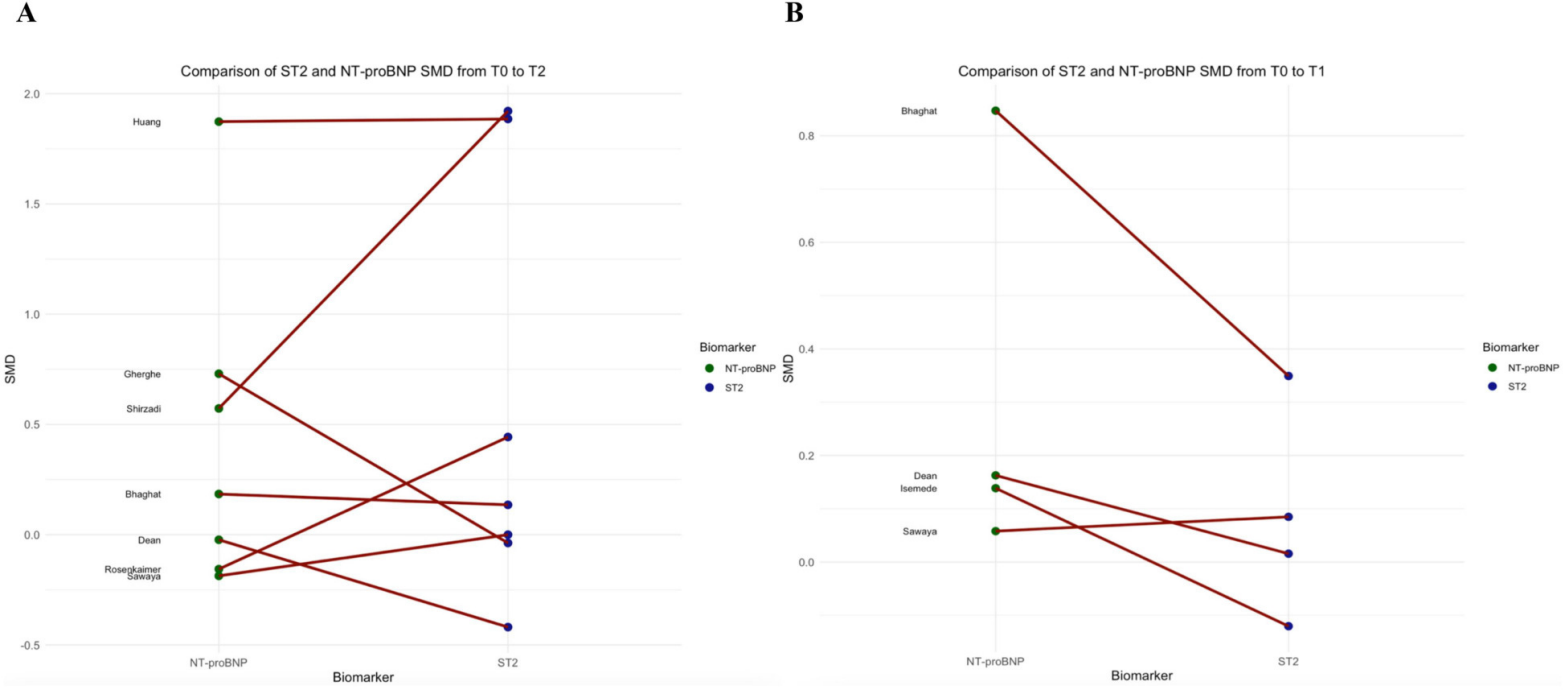
Comparison of NT-proBNP and sST2 standard mean differences between T0 and T2 (panel **A**), and T0 and T1 (panel **B**).

To account for unit of measure heterogeneity, the SMD of sST2, Troponin, and NT-proBNP were calculated between T0 and T2 and T0 and T1. Table 3 summarizes the SMDs and *p*-values which express differences between sST2 and either Troponin or NT-proBNP SMDs. Overall, while the sST2 SMD was not significantly different from Troponin SMD between T0 and T2 (*p* = 0.180, Figure 3A), their SMDs were significantly different between T0 and T1 with Troponin SMD being significantly higher (*p* = 0.027, Figure 3B). A significant difference between sST2 and NT-proBNP was observed neither between T0 and T2 (*p* = 626, Figure 4A) nor between T0 and T1 (*p* = 140, Figure 4B).

**Table 3:** Standard mean difference for sST2, NT-proBNP, and troponin at T0–T2 and T0–T1. *P*-values are expressions of differences between sST2 and NT-proBNP standard mean difference first and between sST2 and Troponin standard mean difference second.

First author	T0–T2					T0–T1				
	sST2	NT-proBNP	*p*-value	Troponin	*p*-value	sST2	NT-proBNP	*p*-value	Troponin	*p*-value
Bhaghat	0.135	0.184	0.626	–	0.180	0.349	0.847	0.140	–	0.027
Dean	−0.419	−0.023		1.035		0.016	0.163		0.943	
Gherghe	−0.0377	0.730		0.243		–	–		–	
Huang	1.885	1.873		–		–	–		–	
Isemede	–	–		–		−0.120	0.139		1.523	
Rosenkaimer	0.443	−0.156		0.228		–	–		–	
Sawaya	0.000	−0.187		1.173		0.085	0.058		1.278	
Shirzadi	1.921	0.573		–		–	–		–	

## Discussion

In this systematic review and meta-analysis of 8 studies including 433 patients affected by cancer, primarily females with breast cancer, treated with cardiotoxic therapies, we explored the role of sST2 as a monitor biomarker for cardiotoxicity. We observed that [i] although a trend towards increased sST2 values between T0 and T2 and a trend towards decreased sST2 values between T1 and T2 was observed, we did not report any significant longitudinal change; [ii] a significant pooled correlation between increased sST2 levels with reduced left ventricular ejection fraction was observed; [iii] while we report a significant higher SMD for Troponin between T0 and T1 compared to sST2, we did not observe any other significant differences in terms of SMD between biomarkers in any of the timings.

The observed trend towards higher sST2 values during follow-up compared to baseline and the trend towards lower sST2 values at follow-up compared to the sample drowning after chemotherapy completion are probably the most interesting findings of our study. The former strengthens the potential role of sST2 in monitoring cancer patients exposed to cardiotoxic therapies. Mechanistically, IL-33 is primarily released by interstitial cardiac cells and plays a protective role by mitigating inflammation, hypertrophy, and fibrosis. In contrast, sST2 acts as a decoy receptor, sequestering IL-33 and attenuating its beneficial effects (23–25). Thus, while IL-33 functions as an anti-inflammatory cytokine with cardioprotective properties, sST2 is produced in response to increased cardiac load and promotes inflammatory activation and fibrosis by inhibiting the actions of IL-33 (26). In light of this, an increase in sST2 during cardiotoxic therapy might be an early sign of a suffering myocardium. By contrast, the latter might be an expression of partial myocardial recovery after chemotherapy completion due to either spontaneous recovery or anti-neurohormonal therapy administration. Specifically, Bhagat et al. reported that despite cardioprotective medications did not impact left ventricular ejection fraction, five patients were started on a beta-blocker, and four patients on an angiotensin-converting-enzyme inhibitor/angiotensin receptor blocker (27). Similarly, Sawaya et al. prescribed beta blockers to one patient who developed cardiotoxicity (28), while Dean et al. did not report if cardioprotective medications were prescribed (29). Accordingly, the medication's cardioprotective effect on sST2 levels might be only hypothesized.

In contrast to our findings, some studies included in our meta-analysis and others excluded for various reasons did not report a correlation between sST2 levels and the development of cardiotoxicity (30). Specifically, a retrospective/prospective study involving breast cancer patients with germline BRCA1/2 mutations and normal left ventricular ejection fraction found no association between sST2 levels and left ventricular ejection fraction decline, regardless of BRCA1/2 status (30). Conversely, the study by Frères et al., which was excluded from our meta-analysis because it only reported sST2 fold changes for patients who exhibited an increase in sST2, without providing absolute values or data for those without an increase, observed significantly higher sST2 levels in patients who developed congestive heart failure, supporting our findings (31). Given these heterogeneous observations, our results should be regarded as exploratory. Further research is needed to analyze biomarkers, including sST2, in a standardized longitudinal framework, directly comparing patients who develop cardiotoxicity with those who do not.

Troponin and natriuretic peptides, such as NT-proBNP, are the most widely routinely used biomarkers to monitor, screen, and diagnose cardiotoxicity (8). However, they have several limitations (32). Despite troponin's predictive and prognostic utility in detecting cardiotoxicity, it is limited by the lack of standardized assays, significant biological variability, inconsistent application of novel decision limits, and reliance on thresholds derived from other clinical contexts, which may compromise their sensitivity and specificity in chemotherapy-induced cardiotoxicity (32). Additionally, its elevations can be influenced by highly prevalent non-cardiac factors in cancer patients, including renal dysfunction and systemic inflammation, complicating its interpretation in oncology patients. Similarly, natriuretic peptides are affected by significant biological variability, susceptibility to confounding by comorbidities such as renal dysfunction, and threshold heterogeneities used in studies (32). Despite these limitations, these are the biomarkers of reference prompting our analysis, which aimed to compare the SMDs of sST2 with troponin and NT-proBNP. The only notable difference was observed during the early phases of cardiotoxic treatment, where troponin demonstrated greater longitudinal changes, likely reflecting its higher sensitivity compared to sST2. Further studies are required to directly compare the longitudinal changes and predictive values of commonly used biomarkers, such as troponin, against sST2.

Managing cancer patients undergoing cardiotoxic therapies is a complex and increasingly important challenge in the field of cardio-oncology. Biomarkers represent just one component of a comprehensive evaluation that should also incorporate cardiovascular risk factors, pre-existing cardiovascular diseases, comorbidities, overall functional status, genetics, and genomics (33–36). Accordingly, given the negative correlation between sST2 and LVEF, sST2 may capture subclinical myocardial injury and functional decline, reinforcing its potential role as an adjunct to existing risk stratification tools in cardio-oncology. In particular, sST2 could serve as a “bonus item” complementing established scores such as the HFA-ICOS risk score, potentially improving the early identification of patients at risk of cardiotoxicity. Continued efforts are needed to develop a tailored approach for cancer patients, identifying those at increased risk and, critically, detecting cardiotoxicity at its earliest stages to facilitate the implementation of precision medicine.

### Limitations

To the best of our knowledge, this is the first meta-analysis to address this important topic. However, this work has limitations that must be acknowledged.

First, we included studies that assessed sST2 values in cancer patients exposed to cardiotoxic therapies. Although several cardiotoxic therapies are used in clinical practice, we focused on anthracycline and HER2 antibodies. Additionally, we included a limited proportion of patients without breast cancer, which limits the generalizability of our results. A direct comparison between patients who develop cardiotoxicity vs. those who do not would have been desirable, and future studies are needed to specifically investigate sST2 trajectories. Second, multiple sST2 assays have been employed, limiting the generalizability of our results. Third, correlations between sST2 and cardiotoxicity were heterogeneous among studies, further limiting the generalizability of our results. Fourth, we had a relatively short follow-up period, which limited the analysis of long-term dynamics of sST2. Biomarkers longitudinal changes analysis on expanded follow-up is expected. Finally, given that we performed a meta-analysis on absolute values without including any outcome's original research manuscripts, a study quality assessment is not feasible.

## Conclusions

This exploratory systematic review and meta-analysis highlighted the potential role of sST2 as a biomarker for cardiotoxicity monitoring during cardiotoxic chemotherapy. Although sST2 levels demonstrated variations across different time points and correlated negatively with left ventricular ejection fraction, these absolute changes were not statistically significant. Additionally, troponin exhibited greater longitudinal changes than sST2, particularly in the early phases of treatment. Despite its promise, the utility of sST2 in this context remains limited by the lack of significant findings and the variability among studies. Future longitudinal studies focusing on patients who develop cardiotoxicity compared to those who do not are warranted. Such research could clarify the clinical value of sST2 in detecting early signs of chemotherapy-induced cardiac dysfunction and guide its potential integration into routine cardio-oncology practice.

## Data Availability

The original contributions presented in the study are included in the article/Supplementary Material, further inquiries can be directed to the corresponding author/s.
