# Supplementary material for: Soluble suppression of tumorigenicity-2 changes during cardiotoxic cancer treatment: a systematic review and meta-analysis
Source: Front Cardiovasc Med. 2025 Nov 11;12:1624023. doi: 10.3389/fcvm.2025.1624023 (PMC12645410; doi:10.3389/fcvm.2025.1624023)
Supplement: Supplementary file 1 [file Supplementaryfile1.docx]

**Supplementary Material**

**Supplementary Tables**

**Supplementary Table 1.** Detailed search strategy.

**Supplementary Table 2.** Definitions of cardiotoxicity for each study.

**Supplementary Table 3.** Methodology of ST2 measurement.

**Supplementary Figures**

**Supplementary Figure 1.** Study selection PRISMA flowchart.

**Supplementary Table 1.**

| (sST2 OR ST2) AND (anthracycline OR cardiotoxicity OR "cardio-oncology" OR cardio oncology OR "cardiovascular toxicity" OR "cardiac toxicity" OR "Chemotherapy induced cardiotoxicity" OR "Chemotherapy-Induced cardiotoxicity") |
| --- |

**Supplementary Table 2**.

| **Bhagat** | Decrease in left ventricular ejection fraction (LVEF) ≥20% when the baseline LVEF is normal or a decrease in LVEF ≥10% when the baseline LVEF is less than the institutional lower limit of normal. |
| --- | --- |
| **Dean** | Not reported. They applied a correlation with LVEF. |
| **Gherghe** | Not reported. They applied a correlation with LVEF. |
| **Huang** | Not reported. They applied a correlation with LVEF. |
| **Rosenkaimer** | Any decrease in LVEF >10% or <50% or increase in GLS >15% or > −16% was defined as cardiac event. |
| **Sawaya** | Either a cardiomyopathy with decreased LVEF, a reduction of LVEF ≥5% to <55% with symp­toms of heart failure (diagnosed by a cardiologist at the site), or an asymptomatic reduction of LVEF ≥10% to <55%. |

**Supplementary Table 3.** Methodology of ST2 measurement.

| **First Author** | **Method of ST2 measurment** |
| --- | --- |
| Bhaghat | Magnetic particle chemiluminescence method using Lidman equipment |
| Dean | Human Magnetic Cardiac Panel A (LUCAM523) |
| Gherghe | Quantikine ELISA kits from R&D Systems, Inc., Minneapolis, MN, USA |
| Huang | Quantikine ELISA kits from R&D Systems, Inc., Minneapolis, MN, USA |
| Isemede | Presage® ST2 assay kit (obtained from Critical Diagnostics San Diego, CA) on Dynex DS2 automated ELISA system (Dynex Technologies, Inc. Chantilly, VA, USA) |
| Rosenkaimer | Quantikine ELISA kits from R&D Systems, Inc., Minneapolis, MN, USA |
| Sawaya | Presage ST2, Critical Diagnostics, San Diego, CA) on an automated enzyme linked–immunosorbent assay platform |
| Shirzadi | Enzyme-Linked Immunosorbent Assay (ELISA) kits (bioassay technology laboratory, China) |

**Supplementary Figure 1**. PRISMA flow diagram of study screening and selection.
